# Nasal polyps show decreased mucociliary transport despite vigorous ciliary beating

**DOI:** 10.1016/j.bjorl.2023.101377

**Published:** 2023-12-15

**Authors:** Thi Nga Nguyen, Yuma Koga, Tetsuro Wakasugi, Takuro Kitamura, Hideaki Suzuki

**Affiliations:** a University of Occupational and Environmental Health, School of Medicine, Department of Otorhinolaryngology-Head and Neck Surgery, Kitakyushu, Japan; b Vinh Medical University, Faculty of Public Health, Vinh City, Vietnam

**Keywords:** Human nasal mucosa, Mucociliary transport velocity, Ciliary beat frequency, Planar cell polarity proteins, Nasal polyp

## Abstract

**Objective:**

Mucociliary transport function in the airway mucosa is essential for maintaining a clean mucosal surface. This function is impaired in upper and lower airway diseases. Nasal polyps are a noticeable pathological feature that develop in some of the patients with chronic rhinosinusitis. Like ordinary nasal mucosae, nasal polyps have a ciliated pseudostratified epithelium with vigorous ciliary beating. We measured *ex vivo* Mucociliary Transport Velocity (MCTV) and Ciliary Beat Frequency (CBF) and explored the expressions of Planar Cell Polarity (PCP) proteins in nasal polyps in comparison with turbinate mucosae.

**Methods:**

Inferior turbinates and nasal polyps were surgically collected from patients with chronic rhinosinusitis. *Ex vivo* MCTV and CBF were measured using a high-speed digital imaging system. Expressions of PCP proteins were explored by fluorescence immunohistochemistry and quantitative RT-PCR.

**Results:**

The MCTV of nasal polyps was significantly lower than that of the turbinates (7.43 ± 2.01 vs. 14.56 ± 2.09 μm/s; *p* = 0.0361), whereas CBF did not differ between the two tissues. The MCTV vector was pointed to the posteroinferior direction in all turbinates with an average inclination angle of 41.0 degrees. Immunohistochemical expressions of Dishevelled-1, Dishevelled-3, Frizzled3, Frizzled6, Prickle2 and Vangl2 were lower in the nasal polyps than in the turbinates. Confocal laser scanning microscopy showed that Frizzled3 was localized along the cell junction on the apical surface. The expression levels of mRNAs for Dishevelled-1, Dishevelled-3 and Frizzled3 in the nasal polyps were also decreased in comparison with the turbinates.

**Conclusion:**

These results indicate that muco ciliary transport in nasal polyps is impaired although vigorous ciliary beating is maintained, and that the impairment may be caused by a decrease in Dishevelled/Frizzled proteins and resultant PCP disarrangement.

**Level of evidence:**

Level 3.

## Highlights

• Mucociliary transport velocity of nasal polyps was lower than that of turbinates.
• Ciliary beat frequency did not differ between the two tissues.
• Expression of Planar Cell Polarity (PCP) proteins was decreased in nasal polyps.
• Lowered mucociliary transport in nasal polyps may be due to disarranged PCP.

## Introduction

Mucociliary transport is an essential function in the airway mucosa to eliminate any foreign particles, allergens, and pathogens that may be adhering to the mucosal surface. The mucociliary transport function is mainly dependent on ciliary beating and the physical properties of the surface mucus. This function is known to be impaired in pathological conditions of the upper and lower airways, such as chronic sinusitis, allergic rhinitis, bronchial asthma, bronchiectasis, chronic obstructive pulmonary disease, and cystic fibrosis.^1^, ^2^, ^3^, ^4^

Nasal polyps are inflammatory outgrowths of the sinonasal mucosa that are noticeable pathological changes in some of the patients with chronic allergic/nonallergic rhinosinusitis.^5^ Like the ordinary nasal mucosa, nasal polyps have ciliated pseudostratified epithelia.^6^ Interestingly, our recent study showed that nasal polyp tissue exhibited vigorous ciliary beating and the baseline Ciliary Beat Frequency (CBF) of this tissue was not decreased compared to that of the turbinate mucosae.^7^ We conjectured that, although the CBF is maintained at the same level in nasal polyps as in the ordinary nasal mucosa, the temporal and/or spatial coordination of the ciliary movement may be impaired in the former, which would lead to a decline in the mucociliary clearance function of this tissue.

In the present study, we measured the *ex vivo* Mucociliary Transport Velocity (MCTV) of nasal polyps in comparison with that of the inferior turbinate mucosae, and also explored the expressions of Planar Cell Polarity (PCP) proteins, which are responsible for coordinating cell polarization in epithelial tissues.

## Methods

### Patients and sample collection

A total of 34 chronic rhinosinusitis patients with hypertrophic rhinitis and/or nasal polyps were enrolled in this study. They consisted of 25 males and 9 females, aged 17–81 years, with an average age of 48.8 years. Total and/or specific serum IgE levels were positive in 26 patients (76.5%). Specific serum IgE levels were measured for house dust mites, Japanese cedar pollen, cypress pollen, orchard grass pollen, short ragweed pollen, timothy grass pollen and *Aspergillus*, which are major airborne allergens in Japan. Six patients had bronchial asthma.

The patients underwent endoscopic sinonasal surgery including turbinectomy and/or nasal polypectomy under general anesthesia. In turbinectomy, the inferior turbinate bone was resected together with the lateral mucosa of the turbinate. The collected inferior turbinates and nasal polyps were immediately soaked in O_2_-saturated Hank’s balanced salt solution (HBSS; 8000 (in mg/L) NaCl, 400 KCl, 350 NaHCO_3_, 140 CaCl_2_, 100 MgCl_2_·6H_2_O, 100 MgSO_4_·7 H_2_O, 60 KH_2_PO_4_, 47.8 Na_2_HPO_4_, and 1000 glucose) and thoroughly washed with HBSS to remove surface mucus. The lateral mucosae of the collected turbinates were separated from the underlying bone with surgical scissors. The turbinate mucosae and nasal polyps were then subjected to the following process.

### Preparation of mucosal pieces for video recording

The turbinate and nasal polyp mucosae were cut into slender strips, including the mucosal surface along the long axis, using a razor blade. Two mucosal strips lying at right angles to each other were prepared from each sample. In the turbinate mucosae, the two strips were cut parallel to the horizontal and vertical axes, respectively. The mucosal strips were immediately immersed in O_2_-saturated HBSS and transferred into a 20 × 6 × 1-mm test chamber filled with the same solution containing 0.05% (v/v) India ink as a tracer. Mucociliary beating/transport was observed under a Nikon Eclipse 80i phase-contrast light microscope (Nikon, Tokyo, Japan) equipped with a high-speed digital imaging system HAS-U1 (DITECT, Tokyo, Japan). Four recordings of 2–3 s each were made at 60-s intervals at a speed of 200 frames/s and analyzed by HAS-XViewer Camera Memory ver. 1.2.12 (DITECT). All procedures were performed at room temperature (approx. 24 °C) and completed within 3 h after sample collection.

### Measurement of mucociliary transport velocity (MCTV)

The MCTV of each mucosal strip was determined by measuring the distance traveled of India ink microparticles per unit time. The actual MCTV was calculated by the following formula: Actual MCTV = $\sqrt{{{\text{M}\text{C}\text{T}\text{V}}_{\text{x}}}^{2}+{{\text{M}\text{C}\text{T}\text{V}}_{\text{y}}}^{2}\;}$, where MCTV_x_ and MCTV_y_ are the MCTVs along the paired mucosal strips lying at right angles to each other. Then, the actual MCTVs of the right and left turbinates were averaged in each patient.

### Measurement of ciliary beat frequency (CBF)

The number of ciliary beats was counted manually by checking the video in a slow replay mode. CBF was measured at three different sites of each mucosal strip. The CBF value in each patient was determined by averaging 12 measurements (3 sites × 2 mucosal strips × both sides).

### Fluorescence immunohistochemistry

Fluorescence immunohistochemistry was performed as described previously.^7^ Primary antibodies were rabbit anti-human Dishevelled-1 polyclonal antibody (PAJ540Hu01; Cloud-Clone, Houston, TX, USA), rabbit anti-human Dishevelled-3 polyclonal antibody (PAL453Hu01; Cloud-Clone), rabbit anti-human Frizzled3 polyclonal antibody (CSB-PA882067ESR2HU; Cusabio, Houston, TX, USA), rabbit anti-human Frizzled6 polyclonal antibody (G260; Assay Biotech, Fremont, CA, USA), mouse anti-human Prickle1 monoclonal antibody (sc-393034; Santa Cruz Biotechnology, Dallas, TX, USA), rabbit anti-human Prickle2 polyclonal antibody (CSB-PA773783ESR1HU; Cusabio), mouse anti-human Vangl1 monoclonal antibody (sc-166844; Santa Cruz Biotechnology), and mouse anti-human Vangl2 monoclonal antibody (sc-515187; Santa Cruz Biotechnology). The antibodies were used at dilutions of 1:50.

### Confocal laser scanning microscopy

The specimens were fixed with 4% paraformaldehyde in Phosphate-Buffered Saline (PBS) at 4 °C overnight. The fixed samples were washed with PBS with 0.3% Triton X-100 (PBST), treated with 1.5% normal goat serum in PBST for 1 h, and incubated with rabbit anti-human Frizzled3 polyclonal antibody (CSB-PA882067ESR2HU; Cusabio) at 4 °C overnight. The primary antibody was used at a dilution of 1:50 in PBST containing 0.5% Bovine Serum Albumin (BSA). After a brief rinse with PBST, the samples were reacted at room temperature for 2 h with a secondary antibody, Alexa Fluor 488-conjugated goat anti-rabbit IgG (Invitrogen), diluted 1:1000 in PBST containing 0.5% BSA. After washing with PBS, the samples were placed with the apical surface downward in a thin-glass-bottomed Petri dish filled with PBS, gently pressed onto the bottom by metal weights, and examined under a Carl Zeiss LSM880 confocal laser-scanning microscope (Carl Zeiss).

The light source, an argon laser, was coupled to an Axio Observer7 inverted microscope (Carl Zeiss). Excitation wavelength was 488 nm. Laser power was reduced to 1.0% by a neutral density filter. An objective was Plan-Apochromat 40× with oil immersion. Emitted fluorescence was detected by a photomultiplier and displayed as a 512 × 512 pixel resolution image using Carl Zeiss ZEN 2 software.

### Quantitative reverse transcription-polymerase chain reaction (qRT-PCR)

Preparation of Total RNA and qRT-PCR were performed as described previously.^7^ The TaqMan Gene Expression Assays for Dishevelled-1 (*DVL1*; assay identification number Hs00182896_m1), *Dishevelled-3* (*DVL3*; assay identification number Hs00610263_m1), *Frizzled3* (*FZD3*; assay identification number Hs00907280_m1), *Frizzled6* (*FZD6*; assay identification number Hs00171574_m1), *Prickle1* (*PRICKLE1*; assay identification number Hs01055551_m1), *Prickle2* (*PRICKLE2*; assay identification number Hs00291033_s1), *Vangl1* (*VANGL1*; assay identification number Hs01572998_m1), *Vangl2* (*VANGL2*; assay identification number Hs00393412_m1), and Glyceraldehyde-3-Phosphate Dehydrogenase (*GAPDH*) as a housekeeping gene (assay identification number Hs99999905_m1) were purchased from Applied Biosystems (Foster City, CA, USA). The measured Threshold Cycle (C_T_) was normalized by subtracting the C_T_ for *GAPDH* of each sample from those for the target mRNAs. From the obtained ΔC_T_ values, the ratio of the target mRNA to *GAPDH* mRNA was calculated by the following formula: Target mRNA/GAPDH mRNA ratio = 2^-ΔC_T_^.

### Statistical analysis

Data are expressed as means ± SEM. Statistical analysis was performed with BellCurve for Excel Statistics (Social Survey Research Information Co., Tokyo, Japan). Differences between two groups were analyzed by the two-tailed Student *t*-test; *p*-values < 0.05 were considered significant.

## Results

The turbinates and polyps were collected from 21 and 18 patients, respectively (Both tissues were collected from 5 patients). Nine turbinates and 6 polyps were subjected to MCTV/CBF measurements. Four each of turbinates and polyps were subjected to confocal laser scanning microscopy. qRT-PCR was performed in 8 each of turbinates and polyps, of which 6 each were also subjected to fluorescence immunohistochemistry.

**Figure 1:**
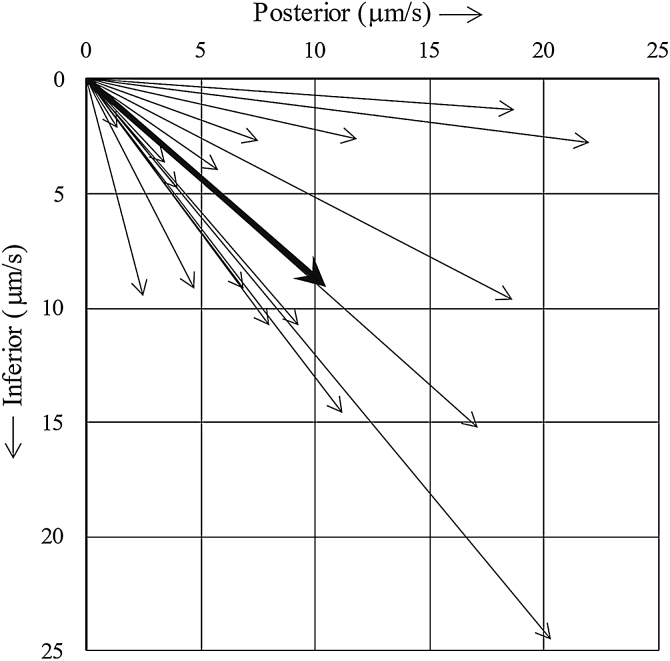
MCTV vector of each turbinate. The MCTV vector pointed to the posteroinferior direction in all turbinates (thin arrows). The average inclination angle was 41.0 degrees (thick arrow). MCTV, Mucociliary Transport Velocity.

The mean actual MCTV of nasal polyps was 7.43 ± 2.01 μm/s (n = 6), which was significantly less than that of the turbinate mucosae (14.56 ± 2.09 μm/s (n = 9), *p* = 0.0361). On the other hand, CBF was not statistically different between the two tissues (7.47 ± 0.13 Hz (turbinate, n = 9) vs. 7.83 ± 0.23 Hz (nasal polyp, n = 6), *p* = 0.1699), consistent with our previous observation.^7^ Fig. 1 shows the MCTV vector of each turbinate. The vector was pointed toward the posteroinferior direction in all turbinates with an average inclination angle of 41.0 degrees.

**Figure 2:**
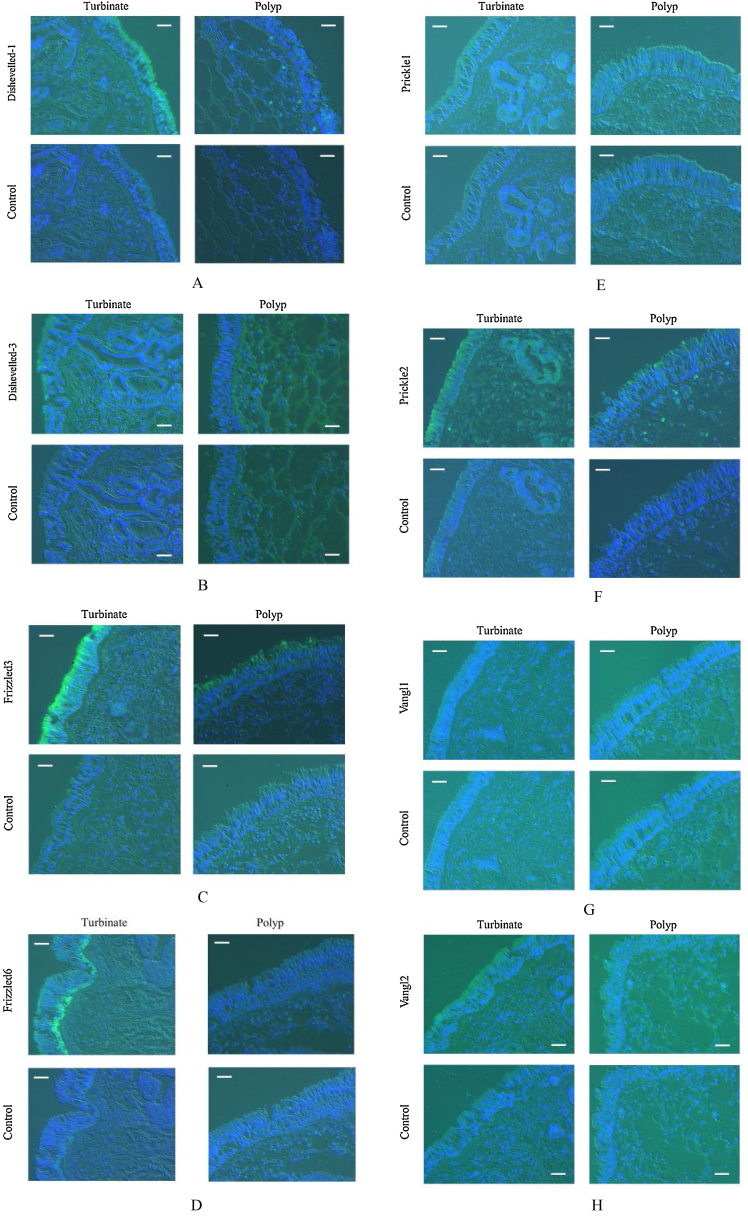
Photomicrographs of fluorescence immunohistochemical staining of the turbinate and nasal polyp for the PCP proteins. Green and blue colors express the fluorescence of Alexa Fluor 488 and DAPI, respectively. Scale bar = 20 μm. (A) Dishevelled-1. Moderate fluorescence is observed on the surface of the turbinate mucosa, whereas the nasal polyp shows no immunoreactivity. (B) Dishevelled-3. Weak fluorescence is observed on the surface of the turbinate mucosa, whereas the nasal polyp shows no immunoreactivity. (C) Frizzled3. Strong and weak fluorescence are seen on the surface of the turbinate mucosa and nasal polyp, respectively. (D) Frizzled6. Weak and moderate fluorescence are observed on the surface and in the basal layer of the turbinate epithelium, respectively, while the nasal polyp shows no immunoreactivity. (E) Prickle1. There is no immunoreactivity in either the turbinate mucosa or nasal polyp. (F) Prickle2. Moderate and weak fluorescence are seen on the surface of the turbinate mucosa and nasal polyp, respectively. (G) Vangl1. There is no immunoreactivity in either the turbinate mucosa or nasal polyp. (H) Vangl2. Weak fluorescence is observed on the surface of the turbinate mucosa, whereas the nasal polyp shows no immunoreactivity.

**Figure 3:**
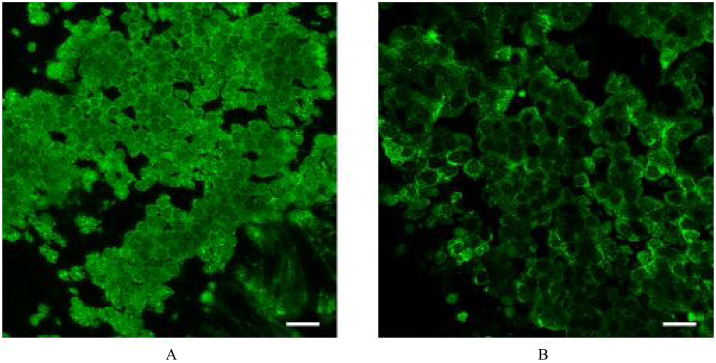
Confocal laser scanning micrographs of the turbinate (A) and nasal polyp (B) stained for Frizzled3. The honeycomb pattern of fluorescence indicates that Frizzled3 is localized along the cell junction on the apical surface. Scale bar = 20 μm.

Fig. 2 represents photomicrographs of fluorescence immunohistochemical staining of the turbinate mucosae and nasal polyp tissue for 8 different PCP proteins; Dishevelled-1, Dishevelled-3, Frizzled3, Frizzled6, Prickle1, Prickle2, Vangl1, and Vangl2. Moderate and weak fluorescence was observed for Dishevelled-1 and Dishevelled-3, respectively, on the surface of the turbinate mucosa, whereas the nasal polyps showed no immunoreactivity for these proteins (Fig. 2 A and B). For Frizzled3, strong and weak fluorescence was seen on the surface of the turbinate mucosa and nasal polyp, respectively (Fig. 2C). Frizzled6 showed weak and moderate fluorescence on the surface and in the basal layer of the turbinate epithelium, respectively, but no immunoreactivity was detected in the nasal polyps (Fig. 2D). For Prickle2, moderate and weak fluorescence was seen on the surface of the turbinate mucosa and nasal polyp, respectively (Fig. 2F). For Vangl2, weak fluorescence was observed on the surface of the turbinate mucosa, whereas the nasal polyp showed no immunoreactivity (Fig. 2H). No immunoreactivity was observed in either the turbinate mucosa or nasal polyp for Prickle1 or Vangl1 (Fig. 2 E and G). We next performed confocal laser scanning microscopy for Frizzled3, whose fluorescence was most intense among the 8 PCP proteins. Confocal laser scanning micrographs demonstrated the honeycomb pattern of fluorescence for Frizzled3, indicating that this protein is localized along the cell junction on the apical surface (Fig. 3). The results of immunohistochemistry are summarized in Table 1.

**Table 1:** Results of immunohistochemistry and qRT-PCR.

	Immunohistochemistry		qRT-PCR (Ratio of target mRNA/*GAPDH* mRNA)		
	Turbinate	Polyp	Turbinate	Polyp	
Dishevelled1	++	–	0.201 ± 0.048	0.044 ± 0.012	(*p* = 0.0065)
Dishevelled3	+	–	0.564 ± 0.064	0.219 ± 0.061	(*p* = 0.0016)
Frizzled3	+++	+	0.299 ± 0.037	0.136 ± 0.024	(*p* = 0.0021)
Frizzled6	++	–	0.213 ± 0.110	0.123 ± 0.040	(*p* = 0.4540)
Prickle1	–	–	0.041 ± 0.005	0.028 ± 0.007	(*p* = 0.1782)
Prickle2	++	+	0.419 ± 0.366	0.075 ± 0.037	(*p* = 0.3651)
Vangl1	–	–	0.135 ± 0.052	0.148 ± 0.048	(*p* = 0.8621)
Vangl2	+	–	0.214 ± 0.110	0.072 ± 0.021	(*p* = 0.2243)

-, +, ++ and +++ indicate no, weak, moderate, and strong fluorescence, respectively.

The expression level of mRNA for each of the 8 PCP proteins, *DVL1*, *DVL3*, *FZD3*, *FZD6*, *PRICKLE1*, *PRICKLE2*, *VANGL1*, and *VANGL2*, is listed in Table 1. The expression levels of *DVL1*, *DVL3* and *FZD3* mRNAs were significantly lower in the nasal polyps than in the turbinates, while those of *FZD6*, *PRICKLE1*, *PRICKLE2*, *VANGL1* and *VANGL2* mRNAs were not statistically different between the two tissues.

## Discussion

The present study showed that mucociliary transport is suppressed even though active mucociliary beating is maintained in nasal polyps compared to turbinate mucosae. Our study also revealed decreased immunohistochemical expressions of the PCP proteins, Dishevelled-1, Dishevelled-3, Frizzled3, Frizzled6, Prickle2 and Vangl2 in the nasal polyp compared to the turbinate mucosa. The expression levels of Dishevelled-1, Dishevelled-3 and Frizzled3 in the nasal polyps were also decreased at the transcriptional level.

Generally, epithelial cells exhibit two-way polarity: polarity along the apical-basal axis and that across the plane of the epithelial sheet. The latter is referred to as PCP, an essential property for regulating epithelial tissue integrity and its developmental process.^8^ The airway epithelial surface is lined with numerous ciliated cells. Each ciliated cell has 200–300 motile cilia on its apical surface.^9^ The cilia are continuously beating to eliminate foreign bodies from the mucosal surface, an important frontline defense mechanism in this tissue. The ciliary beating must be coordinated temporally and spatially to exert effective mucociliary transport function. To generate this coordinated beating, the epithelial cells are arranged in an orderly fashion that makes use of a specific signaling pathway involving the PCP proteins.

Frizzled and Dishevelled are core PCP proteins that constitute the Wnt signaling pathway. There are three main Wnt pathways: the canonical β-catenin-dependent pathway, the noncanonical Wnt/calcium pathway, and the noncanonical Wnt/PCP pathway.^8^, ^10^ The last pathway plays a critical role in PCP formation, and has been investigated in various organs and tissues, such as epithelial tissue,^11^ lung,^10^ cochlea,^12^, ^13^ bile duct,^14^ kidney,^15^, ^16^ vasculature,^17^ neural tube/crest,^12^, ^18^, ^19^, ^20^ gastrula,^18^ and malignant tumors.^21^

Frizzled is a four-span transmembrane protein acting as a Wnt receptor. Dishevelled is a cytoplasmic protein that binds to the intracellular domain of Frizzled. Ten isoforms of Frizzled (Frizzled1-10) and 3 isoforms of Dishevelled (Dishevelled-1, 2 and 3) have been identified in vertebrates. Previous researchers have explored the subcellular localization of these proteins in cultured airway epithelial cells and the *Drosophila* wing epithelium: Frizzled and Dishevelled are eccentrically localized on a specific side of the apical surface of the cells.^22^, ^23^, ^24^

Vangl and Prickle are another pair of core PCP proteins: Vangl is a four-span transmembrane protein, and Prickle is a cytoplasmic protein that binds to the intracellular domain of Vangl.^8^, ^10^ In vertebrates, each protein has two isoforms, Vangl1/2 and Prickle1/2. These molecules are also involved in the noncanonical Wnt/PCP pathway.^25^ Like the Frizzled/Dishevelled complex, the Vangl/Prickle complex is eccentrically localized on the apical surface of the epithelial cells, but on the opposite side of Frizzled/Dishevelled.^8^, ^10^

A prior study reported that defective PCP proteins and ciliary orientation are tightly linked to abnormal ciliary differentiation/formation in chronic rhinosinusitis tissue.^26^ The present results clearly showed that the expression levels of Frizzled and Dishevelled are decreased in nasal polyps at the transcriptional and protein levels, implying the suppression of the Wnt signaling pathways, including the noncanonical Wnt/PCP pathway. The direction of ciliary beating is determined by the microtubule arrangement of the ciliary axoneme. Suppression of the noncanonical Wnt/PCP pathway in nasal polyps would lead to disarrangement of the direction of ciliary beating among ciliated epithelial cells, and consequently, inefficient mucociliary transport function despite the active ciliary beating of each cell. In addition, we showed decreased immunohistochemical expression of Prickle2 and Vangl2 in nasal polyps, and this may also contribute to PCP disarrangement in this tissue.

In cultured epithelial cells, we can observe mucociliary beating and transport movement as the surface image of a top-down view.^27^ If this method had been feasible for the excised tissue samples in our study, we would have been able to directly determine the actual MCTV and the direction of the MCTV vector. However, the thickness of the tissue hindered the clear surface view required for obtaining such a surface image. To overcome this problem, two slender mucosal strips were cut at right angles to each other, and mucociliary beating and transport movement were observed from the side. Then, the actual MCTV and its direction were determined mathematically. The obtained *ex vivo* data in the present study are thought to reflect the *in vivo* phenomenon faithfully and are more valuable than those obtained from cultured cells.

Interestingly, the direction of mucociliary transport was pointed posteroinferiorly in all turbinate mucosa in the present study. Observations in previous *in vivo* human studies using tracers, such as saccharin, radioisotope, dye, and charcoal powder, have indicated that mucociliary transport on the medial surface of the inferior turbinate is directed to the posterior.^28^, ^29^, ^30^, ^31^ We examined the lateral surface mucosa of the turbinate in the present study and obtained a similar result. Through this mucociliary transport function, foreign particles on the turbinate mucosa are conveyed posteriorly, reach the pharynx, and then proceed down the esophagus into the stomach, which is an efficient mechanism in the mucosal defense of the nose. Signaling pathways that determine the PCP direction, and thereby mucociliary transport direction, are of interest in connection with the frontline defense mechanism in the upper airway. Because the inferior turbinate shows almost uniform and steady shape irrespective of different individuals, it was feasible to determine the anatomical direction of the tissue. On the other hand, nasal polyps exhibit various shapes/sizes and protrude to various directions, and therefore, it was unfeasible to clearly determine the anatomical mucociliary transport direction of this tissue.

In regard of immunohistochemistry, we encountered the same difficulty as in the observation of mucociliary movement. In previous studies, immunohistochemical localization of PCP proteins was demonstrated by surface images of a top-down view.^26^, ^32^ Vladar et al.^26^ examined the immunohistochemical expressions of Vangl1 and Prickle2 in cultured tracheal/nasal epithelial cells. Generally, the cultured epithelial cell sheet is filmy and completely flat and it is relatively easy to obtain a plane surface image. Kunimoto et al.^32^ examined the immunohistochemical expression of Vangl1 in the mouse tracheal epithelium. This tissue is also thin and filmy and allow excitation/emission lights pass through the tissue. On the other hand, human nasal turbinate/polyp mucosae are much thicker with uneven surfaces. Because of such attributes of the present samples, it was very difficult to obtain an immunohistochemical surface image, like in the observation of mucociliary movement. In order to overcome this difficulty again, we performed confocal laser scanning microscopy, as in the study by Vladar et al.,^26^ and obtained immunohistochemical surface images that demonstrated the honeycomb pattern of fluorescence for Frizzled3 (Fig. 3), clearly indicating that this protein is localized along the cell junction on the apical surface. However, asymmetric localization was not identified. Vladar et al. also failed to identify asymmetric localization of VANGL1 in human rhinosinusitis epithelia, whereas they observed the asymmetry in normal human sinonasal epithelia.^26^ In the present study, we did not examine healthy control tissues. It is ethically difficult to obtain normal human nasal mucosa of healthy subjects in our institution. Because of this restriction, we had to use nasal mucosa collected from chronic rhinosinusitis patients with hypertrophied turbinate as a control sample. Although the medial side of the hypertrophied turbinate histologically exhibits irreversible changes such as submucosal fibrosis, the lateral mucosa of the turbinate microscopically retains normal fundamental structures.^33^ Based on this observation, we deemed that the influence of the pathological change of the turbinate mucosa was minimal in the present study. Nevertheless, we have to admit that this tissue is still inflamed and may have aberrant localization of PCP proteins. Actually, we did not identify asymmetric localization of Frizzled3 in confocal laser scanning microscopy. This issue is one of the limitations of the present study and remains to be addressed using normal human nasal mucosa in a future study.

## Conclusions

We investigated *ex vivo* mucociliary transport in nasal polyps and turbinate mucosae. The MCTV was significantly less in nasal polyps than in the turbinates despite the similar CBF of the two tissues. Immunohistochemical expressions of the PCP proteins, Dishevelled-1, Dishevelled-3, Frizzled3, Frizzled6, Prickle2 and Vangl2 were lower in the nasal polyps than in the turbinate mucosae. The expression levels of Dishevelled-1, Dishevelled-3 and Frizzled3 in the nasal polyps were also decreased at the transcriptional level. These results suggest that the decreased MCTV in the nasal polyps may be due to impaired PCP. Suppression of the Wnt pathway, which involves the PCP proteins, is likely to play a key role in the pathogenesis of the impaired mucociliary transport of nasal polyps. This point remains for further investigation.

## Ethics approval

Written informed consent was obtained from all the patients enrolled in the study. The study was approved by the institutional review board of the University of Occupational and Environmental Health (UOEHCRB19-014) and conducted in accordance with the World Medical Association Declaration of Helsinki. Two of the enrolled patients were under 20-years of age. In these patients, informed assent of the patients and informed consent of their parent(s) were obtained according to the protocol of the institutional review board.

## Funding

This study was supported by a Grant-in-Aid for Scientific Research (C) (no. 19K09879; 2019‒2022) to H.S. from the Japan Society for the Promotion of Science.

## Conflicts of interest

The authors declare no conflicts of interest.
